# Lung Cryobiopsy Outside of the Operating Room: A Safe Alternative to Surgical Biopsy

**DOI:** 10.1177/15569845211034506

**Published:** 2021-08-01

**Authors:** Vanessa Menezes, Juan Carlos Molina, Clare Pollock, Philippe Romeo, Julie Morisset, Pasquale Ferraro, Edwin Lafontaine, Jocelyne Martin, Basil Nasir, Charles Leduc, Moishe Liberman

**Affiliations:** 1 5622 Division of Thoracic Surgery, CHUM Endoscopic Tracheo-bronchial and Oesophageal Centre, Centre Hospitalier de l’Université de Montréal, Québec, Canada; 2 Department of Pathology, Centre Hospitalier de l’Université de Montréal, Québec, Canada; 3 Division of Pulmonology, Centre Hospitalier de l’Université de Montréal, Québec, Canada

**Keywords:** cryobiopsy, surgical lung biopsy, interstitial lung disease

## Abstract

**Objective:**

Transbronchial lung cryobiopsy (TBLC) is a promising technique that can provide a histologic diagnosis in interstitial lung diseases (ILD) and is an alternative to surgical lung biopsy. The main concerns with the procedure are safety and diagnostic accuracy. The technique is applicable in patients unable to undergo surgical biopsy due to severe comorbidities or when patient transport to the operating room is dangerous. This study reports the initial experience with TBLC on a thoracic surgical service as a first attempt at diagnosis in patients with diffuse parenchymal lung diseases (DPLD).

**Methods:**

Between May 2018 and July 2020, 32 patients underwent TBLC using bedside flexible bronchoscopy for suspected ILD on a thoracic surgical endoscopy service. Retrospective evaluation of the procedure details, complications, and diagnostic yield were analyzed and reported.

**Results:**

A total of 89 pathological samples were obtained (mean 2.8 per patient). Pneumothorax and minor bleeding occurred in 25% and 16.7% of patients, respectively. Sixty-seven percent of complications occurred with use of the 2.4 mm cryoprobe (*P* = 0.036). Concordance between the histologic diagnosis and final clinical diagnosis was observed in 62.5% of patients and the pathology guided the final treatment in 71% (*P* = 0.027) with Kappa-concordance of 0.60 (*P* < 0.001).

**Conclusions:**

Cryobiopsy is becoming part of the diagnostic evaluation in patients with indeterminate DPLD or hypoxemic respiratory failure. TBLC is easy to perform and has a favorable safety profile. Thoracic specialists should consider adding TBLC to their procedural armamentarium as a first option for patients with indeterminate PLD.

**Central Message**

Transbronchial lung cryobiopsy is easy to perform and safe as an alternative to surgical biopsy for diagnosis of interstitial lung diseases. The technique is applicable in patients who are unable to undergo surgical biopsy and should be considered by thoracic specialists.

## Introduction

Transbronchial lung cryobiopsy (TBLC) is gaining popularity as a safe and effective alternative to surgical lung biopsy for the diagnosis of interstitial lung diseases (ILD). The technique has been adopted into clinical practice by pulmonologists in many centres and emerging guidelines are including this approach in the multidisciplinary evaluation for the diagnosis and management of diffuse parenchymal lung diseases (DPLD).^1-3^ TBLC also plays a role in acute and subacute lung infiltrates associated with hypoxemic respiratory failure and detection of acute lung rejection in transplant recipients.^4-6^

**Fig. 1:**
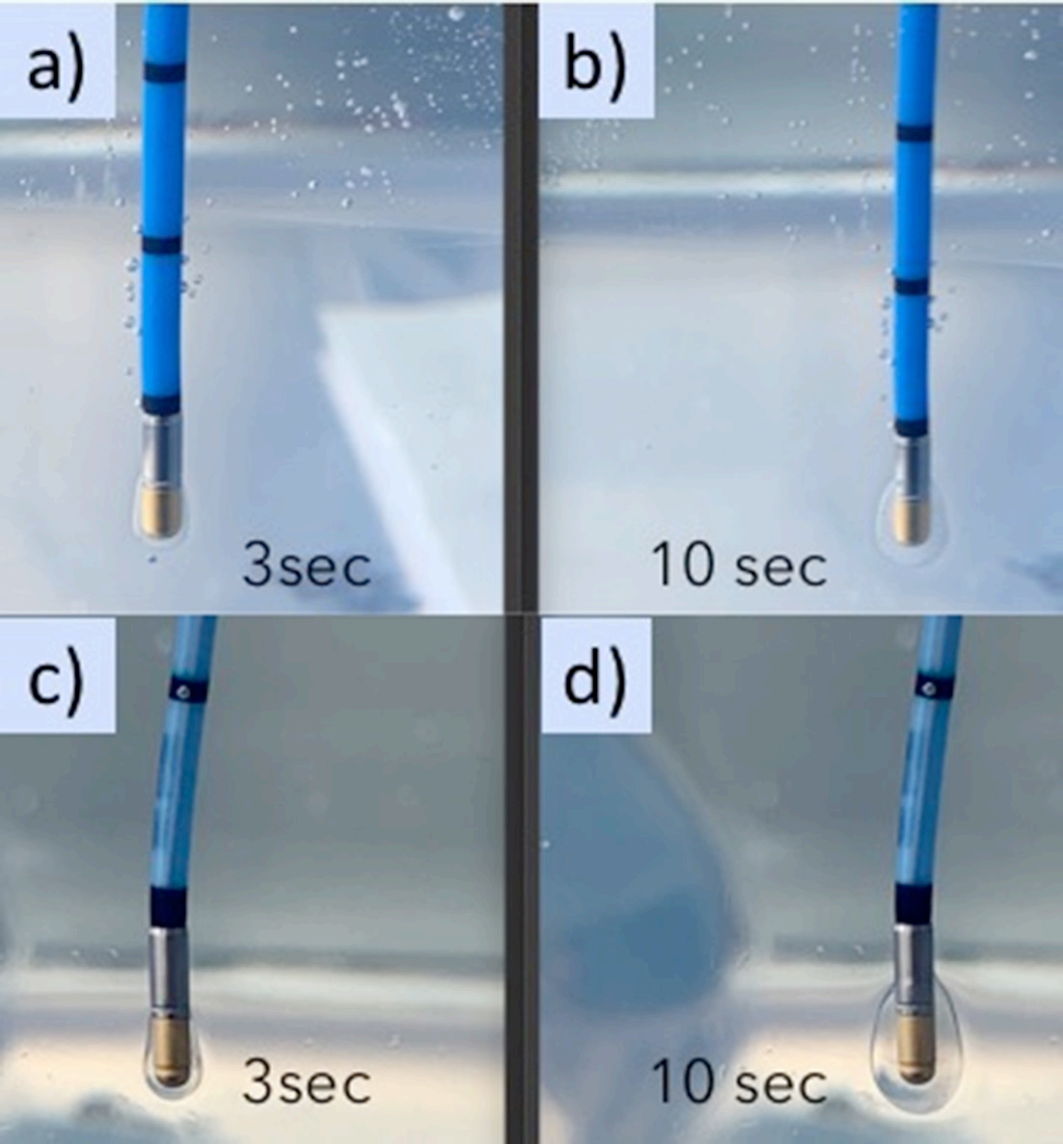
Cryoprobes with associated ice balls; 1.9 mm ERBE cryoprobe (Marietta, GA, USA) activated for (**a**) 3 s and (**b**) 10 s with nitrous oxide gas; 2.4 mm ERBE cryoprobe activated for (**c**) 3 s and (**d**) 10 s with nitrous oxide gas.

**Fig. 2:**
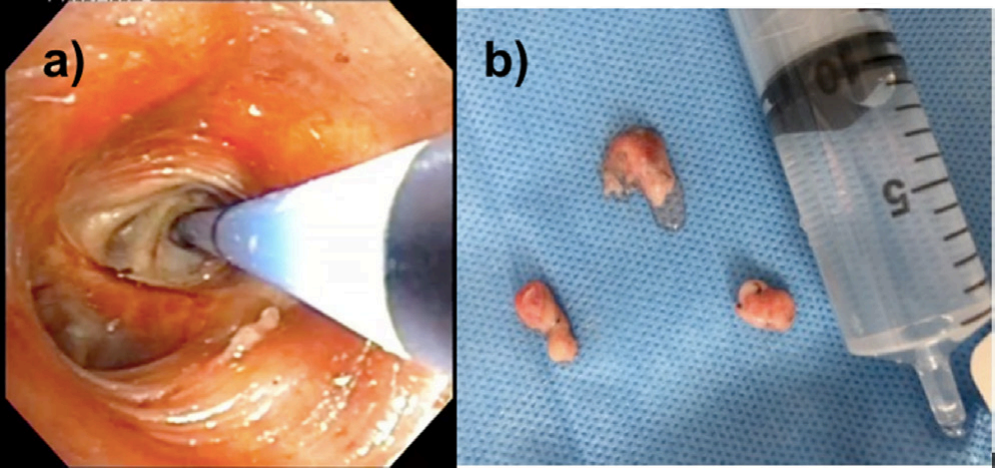
Lung biopsy specimens obtained using transbronchial lung cryobiopsy: (**a**) airway 1.9 mm probe insertion guided by flexible bronchoscopy; (**b**) specimen size.

Cryobiopsy is a minimally invasive technique performed with a flexible or rigid bronchoscope under deep sedation or general anesthesia. This endoscopic intervention can be performed as an outpatient procedure and consists of introducing a flexible cryoprobe through the airway with or without fluoroscopic guidance. The principle of this procedure consists of rapid expansion of compressed nitrous oxide or carbon dioxide gas at high flow rates at the tip of the probe, freezing the surrounding tissue over several seconds (Fig. 1). This causes probe adherence and the lung tissue is then avulsed with a quick pullback movement.^3^ The frozen lung specimens obtained are larger (Fig. 2) than the conventional transbronchial forceps biopsies and provide the pathologist with a specimen with more preserved alveolar structure and less crush artifact, resulting in better quality specimens for histologic analysis.^7^

Surgical lung biopsy (SLB) is still the gold-standard technique to obtain lung tissue specimens for interstitial lung disease (diagnostic yield of 95%). However, SLB has been associated with significant in-hospital mortality (16% for nonelective patients, 1.7% for elective patients, overall 6.4%) and higher costs related to the procedure.^8-11^ Even though there is significant variability in transbronchial cryobiopsy safety profiles reported in literature,^12^ overall, it is associated with variable rates of pneumothorax and bleeding, which are the two most frequent complications related to the procedure.^13^ Both commercially available cryoprobes (1.9 mm and 2.4 mm) have been used to perform TBLC. Current guidelines recommend using the therapeutic bronchoscope for TBLC because of the risk of bleeding and less friction in the working channel during the cryoprobe progression to the pleura through the peripheral segments.^3^ However, the therapeutic bronchoscope can be challenging to use in intubated patients with severe respiratory conditions where the larger outer diameter of the bronchoscope further obstructs the airflow during mechanical ventilation. The lack of standardization of the biopsy technique could be related to the difficulty in determining its true safety and accuracy.^14,15^

This descriptive study reports our initial experience with this technique as a first alternative to obtain histologic diagnosis for patients with DPLD in the endoscopy suite and in the intensive care unit (ICU) on a thoracic surgery service.

## Methods

### Study Design and Participants

This study consists of a retrospective, single-centre assessment of TBLC performed by thoracic surgeons on a thoracic surgery service including patients who underwent TBLC between May 2018 and July 2020. Procedures were performed in either the endoscopy suite under moderate sedation or in intubated patients in the ICU by the Thoracic Interventional Endoscopy Service at a tertiary specialized centre. Patients were identified from a prospectively maintained thoracic interventional endoscopy database. The study was approved by the Institutional Review Board (RB 19.319). Patients with suspicious, indeterminate DPLD who were referred to the thoracic endoscopy centre for a lung biopsy were approached for discussion of biopsy options in clinic and TBLC was agreed upon as the best initial option after discussion with the patient, family, ILD specialized pulmonologist, and thoracic radiologist. Patients in the ICU were selected based on hypoxemic respiratory failure of unknown etiology associated with acute or subacute diffuse interstitial infiltrates and elevated surgical risk after multidisciplinary discussion with intensivists, pulmonologists, microbiologists, and chest radiologists. If an inadequate specimen or a nondiagnostic TBLC was obtained, the patient was referred for SLB.

Relevant data including demographics, procedural location, biopsy site, histological results, and outcomes were obtained. Board certified thoracic surgeons and interventional endoscopy fellows under direct staff supervision performed all biopsies. In the endoscopy suite, conscious sedation was achieved using midazolam and fentanyl. Oxygen saturation, pulse, and blood pressure were continuously monitored. For the patients who underwent the TBLC in the ICU through an endotracheal tube, it was assured that a tube of a minimal diameter of 8.5 mm was in place to allow adequate ventilation around the bronchoscope. A flexible therapeutic bronchoscope (Olympus XT160; Olympus America Inc., Centre Valley, PA, USA) and 1.9 mm or 2.4 mm flexible cryoprobes (ERBE, Marietta, GA, USA) were used for the procedures. The cryoprobe was cooled for 3 to 30 s in order to achieve multiple large lung biopsy specimens. An endobronchial balloon blocker (6 Fr) was available for all the patients in case of moderate to severe bleeding. Bleeding was classified as mild if it required only suction, moderate if it required endoscopic interventions (bronchial blocker occlusion, instillation of tranexamic acid), and severe if requiring a surgical procedure, blood transfusion, or admission to ICU for respiratory or hemodynamic stabilization.^9^

We excluded (a) patients who were not able to keep SpO2 ≥92% after conscious sedation or using an FiO2 of 100% in the ICU; (b) patients on anticoagulation, antiplatelet agents, or with a previous history of coagulopathy.

### Procedures

Biopsy sites were chosen based on radiologic findings on high-resolution computed tomography and were preferentially targeted in the segments where the diffuse interstitial infiltrates were more predominant. The cryoprobe was advanced through the distal bronchus until resistance was felt. Then the probe was pulled back 1 or 2 cm, indirectly indicating 10 and 20 mm of distance from the pleura,^7^ respectively, and the cryoprobe was activated by pressing down on the foot pedal activator for 3 to 30 s before being retracted en bloc, out of the airway with the bronchoscope, with the frozen lung tissue attached to the tip of the probe. If a bronchial blocker was used, it was positioned inside the target bronchus before the biopsy was performed and immediately inflated after removal of the bronchoscope from the airway. The cryoprobe was submerged in a solution of room-temperature saline to release the frozen tissue specimen (Fig. 2), following which the bronchoscope was reintroduced into the airway to check for bleeding. All patients underwent chest radiographs immediately following the procedure.

Specimens were fixed in 10% formalin for a minimum of 12 hr, embedded in paraffin, sectioned at 3 microns, and stained with hematoxylin saffron and phloxine. Experienced pulmonary pathologists performed histologic assessment. In cases with adequate tissue, histologic diagnosis was rendered, followed by interdisciplinary case discussion to determine the final clinical-radiologic-pathologic diagnosis. Biopsies that offered specific information that guided clinicians to define appropriate treatment or even contributed to change or suspend ongoing treatment (e.g., antibiotic, antifibrotic, immunosuppressant) were considered as TBLC that “guided treatment.”

### Outcomes

Ability to make a definitive diagnosis using TBLC and evaluation of the procedural details and related complications (bleeding, pneumothorax) were the primary outcomes for analysis. The hypothesis was that cryobiopsy is safe and is associated with a high diagnostic yield, which would decrease SLB necessity in a high percentage of cases.

### Statistical Analysis

Differences between groups were assessed using the nonparametric Mann-Whitney U test and χ^2^ for normally distributed covariates. A *P* value <0.05 was considered statistically significant. The crosstabulation of the TBLC histopathological pattern and final clinical diagnosis of the multidisciplinary consensus are reported with Cohen’s kappa statistic. The Kappa-concordance coefﬁcient and percentage agreement (both with their 95% conﬁdence intervals [CI]) were computed to analyze the match between final histopathological report and final clinical diagnosis. A kappa value equal or less than 0.20 demonstrates poor agreement, 0.21 to 0.40 fair agreement, 0.41 to 0.60 moderate agreement, 0.61 to 0.80 good agreement, and 0.81 to 1.00 excellent agreement.

## Results

A total of 32 patients underwent cryobiopsies for evaluation of DPLD, 15 females and 17 males (mean age: 60.4 ± 15.1 years). Twenty-eight bronchoscopies were performed in the endoscopy suite (87.5%) with patients under conscious sedation and in 4 patients the procedure was performed in the ICU, at bedside, under mechanical ventilation without transferring the patients to the operating room (Table 1).

**Table 1:** Demographics.

	Intensive care unit	Endoscopy suite
Age	60.34 ± 15.07	
≤45	1 (3.2)	5 (15.7)
46 to 65	2 (6.2)	9 (28.1)
>65	1 (3.2)	14 (43.8)
Sex		
Female	0	15 (46.9)
Male	4 (12.5)	13 (40.6)
Total	4 (12.5)	28 (87.5)

Data presented as *n* (%) or mean ± SD.

Eighty-nine TBLC samples were collected (mean = 2.78 specimens obtained per patient) and sent for histologic analysis. The 1.9 mm and 2.4 mm outer diameter probes were randomly used in 16 patients each. Table 2 shows the technical details of the procedure. Most biopsies were performed using 20 s activation times (31.3%) and were performed with a 20 mm distance from the pleura (18 patients, 56.3%). The probe was activated 3 and 4 times (sample repetitions) in 37.5% and 21.9% of patients, respectively. Three fragments of frozen lung tissue were obtained in 37.5% of the cases performed, 2 specimens obtained in 21.9%, and only 1 specimen in 15.6% of cases (Table 2). In almost all patients (*n* = 30), all specimens were taken from the same lobe. There was no statistically significant difference between the performance, postoperative complications, and the requirement for additional intervention based on probe size. Following TBLC performed with a 1.9 mm ERBE probe, 2 patients required pleural catheters for pneumothorax post-intervention and 1 patient required a bronchial blocker for moderate bleeding (after 30 s of freezing time). Three patients developed a pneumothorax drained by a pleural catheter after TBLC performed with the 2.4 mm probe. One of these patients required video-assisted thoracoscopic surgery (VATS) with talc poudrage to treat a prolonged air leak (>7 days). One case also performed with the 2.4 mm ERBE probe required topical application of tranexamic acid for moderate bleeding after TBLC (Table 3). No blood transfusions or acute surgical interventions were required in any of the patients in this study. Three patients were submitted to a SLB after the cryobiopsies (9.3%; Table 3). One of the patients was included in a specific clinical trial where surgical biopsies were mandatory. One patient had inadequate material for pathological analysis for ILD, and the other had a suspicion of idiopathic pulmonary fibrosis by multidisciplinary team discussion and possible indication for lung transplantation. Since the final histological analysis (hypersensitivity pneumonitis) was different from the multidisciplinary suspicion, the multidisciplinary discussion team decided to refer the patient for SLB.

**Table 2:** Technical Details of the Procedure.

	Mean ± SD or *n* (%)
Specimens obtained (*N* = 89)	2.78 ± 1.16
Probe size (mm)	
1.9	16 (50.0)
2.4	16 (50.0)
Distance from pleura (mm)	
10	14 (43.8)
20	18 (56.3)
Freezing time (seconds)	
3	3 (9.4)
5	12 (37.5)
10	4 (12.5)
20	10 (31.3)
30	3 (9.4)
Samples obtained/patient	
1	5 (15.6)
2	7 (21.9)
3	12 (37.5)
4	7 (21.9)
6	1 (3.1)

**Table 3:** Comparison Between 1.9 mm and 2.4 mm Cryoprobes.

	Probe size (mm)		Total	*P* value
	1.9	2.4		
Freezing time (seconds)				0.889
3	1 (3.2)	2 (6.2)	3 (9.3)	
5	8 (25.0)	4 (12.5)	12 (37.5)	
10	1 (3.2)	3 (9.3)	4 (12.5)	
20	3 (9.3)	7 (21.9)	10 (31.2)	
30	3 (9.3)	—	3 (9.3)	
Distance from pleura (mm)				0.483
10	8 (25.0)	6 (18.8)	14 (43.7)	
20	8 (25.0)	10(31.5)	18 (56.2)	
Location				0.668
RUL	3 (9.3)	3 (9.3)	6 (18.8)	
ML	1 (3.2)	—	1 (3.2)	
RLL	7 (21.9)	4 (12.5)	11 (34.4)	
LUL	—	3 (9.3)	3 (9.3)	
LLL	4 (12.5)	5 (15.6)	9 (28.2)	
RUL/RML	—	1 (3.2)	1 (3.2)	
LUL/LLL	1 (3.2)	—	1 (3.2)	
Lobes biopsied				<0.001
1	15 (46.9)	15 (46.9)	30 (93.8)	
2	1 (3.2)	1 (3.2)	2 (6.2)	
Complication				0.257
No	11 (34.4)	7 (21.9)	18 (56.2)	
Yes	5 (15.6)	9 (28.1)	14 (43.8)	
Postoperative complications				0.746
Pneumothorax	1 (3.2)	5 (15.6)	6 (18.8)	
Bleeding	1 (3.2)	3 (9.3)	4 (12.5)	
Pneumothorax + bleeding	3 (9.3)	1 (3.2)	4 (12.5)	
Additional therapy^a^				0.456
No	13 (40.6)	11 (34.4)	24 (75.0)	
Yes	3 (9.3)	5 (15.6)	8 (25.0)	

Abbreviations: LLL, left lower lobe; LUL, left upper lobe; ML, middle lobe; RLL, right lower lobe; RML, right ML; RUL, right upper lobe.

Data presented as *n* (%).

^a^Additional therapy: 1.9 mm probe included 2 patients drained for pneumothorax and 1 patient had bronchial blocker utilized for moderate bleeding; 2.4 mm probe included 3 patients drained for pneumothorax, 1 patient required videothoracoscopy for prolonged air leak, and 1 patient had administration of tranexamic acid for moderate bleeding.

### Diagnosis

**Fig. 3:**
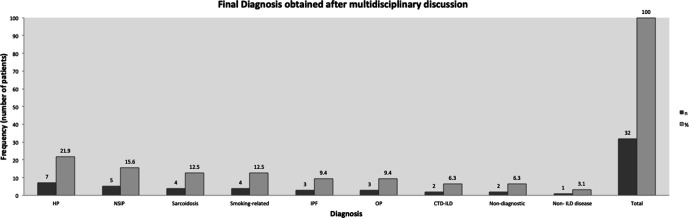
Final diagnosis obtained by multidisciplinary discussion. CTD-ILD, connective tissue disease-associated ILD; HP, hypersensitivity pneumonitis; ILD, interstitial lung disease; IPF, idiopathic pulmonary fibrosis; NSIP, nonspecific interstitial pneumonia; OP, organizing pneumonia pattern.

The concordance rate between final histopathology and final clinical-radiological diagnosis decided upon by the multidisciplinary team was 65.6%, and the pathology obtained through TBLC guided the final treatment in 71.9% of patients (*P* = 0.026; Table 4). The kappa of 0.601 (95% CI: 0.407–0.777, *P* < 0.001) represented a moderate strength of agreement between the final guideline-defined^16^ clinical diagnosis and the pathologic diagnosis. The most frequent diagnosis was nonspecific interstitial pneumonia, followed by usual interstitial pneumonia and organizing pneumonia (Fig. 3).

**Table 4:** TBLC Panel Between Endoscopy Suite and ICU Patients.

	Endoscopy suite *n* = 28	ICU *n* = 4	Total *N* = 32	*P* value
Guided treatment	22 (78.6)	1 (25.0)	23 (71.8)	0.026
Complications	12 (42.8)	2 (50.0)	14 (43.7)	0.736
SLB after procedure	3 (10.7)	—	3 (9.3)	0.492
30-day mortality	—	3 (75.0)	3 (9.3)	<0.001
Concordance between pathology and clinical suspicion by MDT	17 (60.7)	4 (100.0)	21 (65.6)	0.122

Abbreviations: ICU, intensive care unit; MDT, multidisciplinary discussion team; SLB, surgical lung biopsy; TBLC, transbronchial lung cryobiopsy.

Data presented as *n* (%).

### Complications

The 30-day mortality in this series was 9.3%, and all deaths occurred in ICU patients already intubated and undergoing mechanical ventilation prior to the procedure (Table 4). There were no deaths at 30 days in any patients who underwent elective TBLC in an outpatient setting. However, no patient died due to a direct complication of TBLC. The 3 patients who died in the ICU in the 30 days following TBLC were extremely fragile, extremely hypoxemic, and deteriorating in their clinical status prior to TBLC. It was felt that they were too sick to be transported to the operating room for SLB, which is why TBLC was performed.

Pneumothorax was the most common complication and occurred in 18.8% of patients, and bleeding occurred at 12.5% (Table 3). None of the patients required surgical control or rigid bronchoscopy for bleeding related to TBLC. A total of 50% of patients with pneumothorax required pleural drainage by pigtail. One of those cases required talc pleurodesis by VATS for persistent air leak (>7 days). No statistically significant associations were observed between probe size and the risk of complications in this study. One patient experienced moderate bleeding effectively controlled with a 6 Fr endobronchial blocker and continuous suction, and another stopped bleeding following the instillation of tranexamic acid in the airway (Table 3). There were no deaths at 30 days in any patient who underwent elective TBLC in an outpatient setting, nor did any of these patients require intubation or mechanical ventilation following cryobiopsies. No patient required hemodynamic support, blood transfusion, rigid bronchoscopy, or immediate surgical intervention to remedy an acute complication resulting from the TBLC procedure.

## Discussion

This retrospective study reports a contemporary series of TBLC performed on patients outside of the operating room, mostly nonintubated (unless in the ICU). TBLC is a minimally invasive nonsurgical technique associated with lower cost than SLB, can be performed as an outpatient procedure, and can provide good quality specimens with high diagnostic yields.^17-23^ A recently published expert panel guideline recommends that the decision between cryobiopsy and surgical biopsy in specific cases should be based on multidisciplinary decision-making, availability of technologies, and local expertise.^24^

A cost theoretical comparison analysis done in the United Kingdom in 2016 showed that the cost savings of performing a lung cryobiopsy instead of a SLB would be €210 (euros) per patient in the first year and €647 per patient in subsequent years 26. In Spain, for outpatients, the cost of systematic cryobiopsies could save €953.09 per patient. In the same study, when comparing with SLB with 48 hr of hospital admission, the TBLC could save €1,950.29 per patient, featuring cost as an additional advantage of the cryobiopsies for ILD.^25^

The introduction of a new technique is always followed by concerns relating to its safety and accuracy with variability in reported study outcomes, at least in part due to inconsistent study design.^14,15,17-24^ Pneumothorax and massive hemoptysis are the major complications related to lung cryobiopsy. However, acute exacerbation of previous ILD and mortality has also been reported.^24-27^ In this series, there was no mortality and no severe hemoptysis associated with the technique.

Respiratory physicians or thoracic surgeons could perform the procedure without specific training in interventional bronchoscopy. The setting varies with the institution’s experience; it could be performed in the endoscopy suite with or without fluoroscopy guidance. Also, the TBLC could be performed under general anesthesia or conscious sedation with or without an anesthetist.^28^ There is no formal contraindication to proceed with TBLC in stable patients in the ICU eligible for flexible bronchoscopy. Also, cryobiopsy seems to be a wise alternative in the intensive care setting where lung biopsy is recommended, but the patient does not have clinical conditions to be transferred to the operative room. Lung transplant rejection, drug toxicity, and antifibrotic therapy management could be listed in these scenarios. There is no current technical protocol for TBLC, however recently published guidelines recommend specific technical aspects of the procedure to increase the safety profile. However, the lack of standardization of the procedure and weak evidence regarding optimizing the quality of specimens and preventing complications without losing diagnostic accuracy is its main limitations.^28^ A multicentre, prospective Australian trial (COLDICE) published in 2020 showed that cryobiopsy is a valid, first-line, minimally invasive diagnostic tool for ILD patients.^29^ The study showed good agreement between TBLC and SLB for the diagnosis of DPLD; complimentary SLB provided limited additional information for clinicians and is unnecessary in most cases. The pooled outcomes from Australian centres also suggest that the procedure should be performed by an experienced interventionalist using standardized protocols in order to reduce complications.^16,24,30^

This study has various limitations. First, the study consists of a small initial series evaluating the application of the technique of TBLC for DPLD in a nonhomogenous group of patients (28 elective patients under conscious sedation and only 4 nonelective patients with hypoxemic respiratory failure in an ICU context). Second, no comparison SLB group limits the ability to compare complication rates and diagnostic accuracy between the two techniques. Third, our study included ICU patients with more severe respiratory conditions and fragile lung parenchyma that could be linked to a higher risk of pneumothorax and bleeding. However, there is a lack of studies in the literature that evaluate the role of cryobiopsy in this context. In patients with acute respiratory distress syndrome associated with DPLD, TBLC may be an attractive alternative due to the increased risk of surgical complications in this patient group. Future large series of patients with the same severe conditions are required to better analyze the safety and accuracy profiles of TLBC in this context. Lastly, contrary to what is typically recommended when performing SLB for DLPD, we opted to sample only one lobe to avoid multiple sites of trauma during the procedure and decrease the chance of pneumothorax and bleeding. However, this may also decrease the diagnostic yield of TBLC in this population.

## Conclusions

TBLC is feasible, associated with an acceptable risk profile, and accurate for diagnosing ILD. This endoscopic technique is rapidly becoming an important part of the diagnostic armamentarium for patients with indeterminate DPLD and can also be used in hypoxemic respiratory failure patients. Thoracic specialists should consider adding TBLC to their diagnostic skill set as it may replace SLB in the near future, especially for nonsurgical candidates.

## Supplemental Material

Presentation S1 - Supplemental material for Lung Cryobiopsy Outside of the Operating Room: A Safe Alternative to Surgical Biopsy Click here for additional data file. Supplemental material, Presentation S1, for Lung Cryobiopsy Outside of the Operating Room: A Safe Alternative to Surgical Biopsy by Vanessa Menezes, Juan Carlos Molina, Clare Pollock, Philippe Romeo, Julie Morisset, Pasquale Ferraro, Edwin Lafontaine, Jocelyne Martin, Basil Nasir, Charles Leduc and Moishe Liberman in Innovations: Technology and Techniques in Cardiothoracic and Vascular Surgery
