# Supplementary material for: Lung Cryobiopsy Outside of the Operating Room: A Safe Alternative to Surgical Biopsy
Source: Innovations (Phila). 2021 Aug 1;16(5):463–9. doi: 10.1177/15569845211034506 (PMC8637350; doi:10.1177/15569845211034506)
Supplement: Presentation S1 - Supplemental material for Lung Cryobiopsy Outside of the Operating Room: A Safe Alternative to Surgical Biopsy [file sj-pptx-1-inv-10.1177_15569845211034506.pptx]

## Slide 1
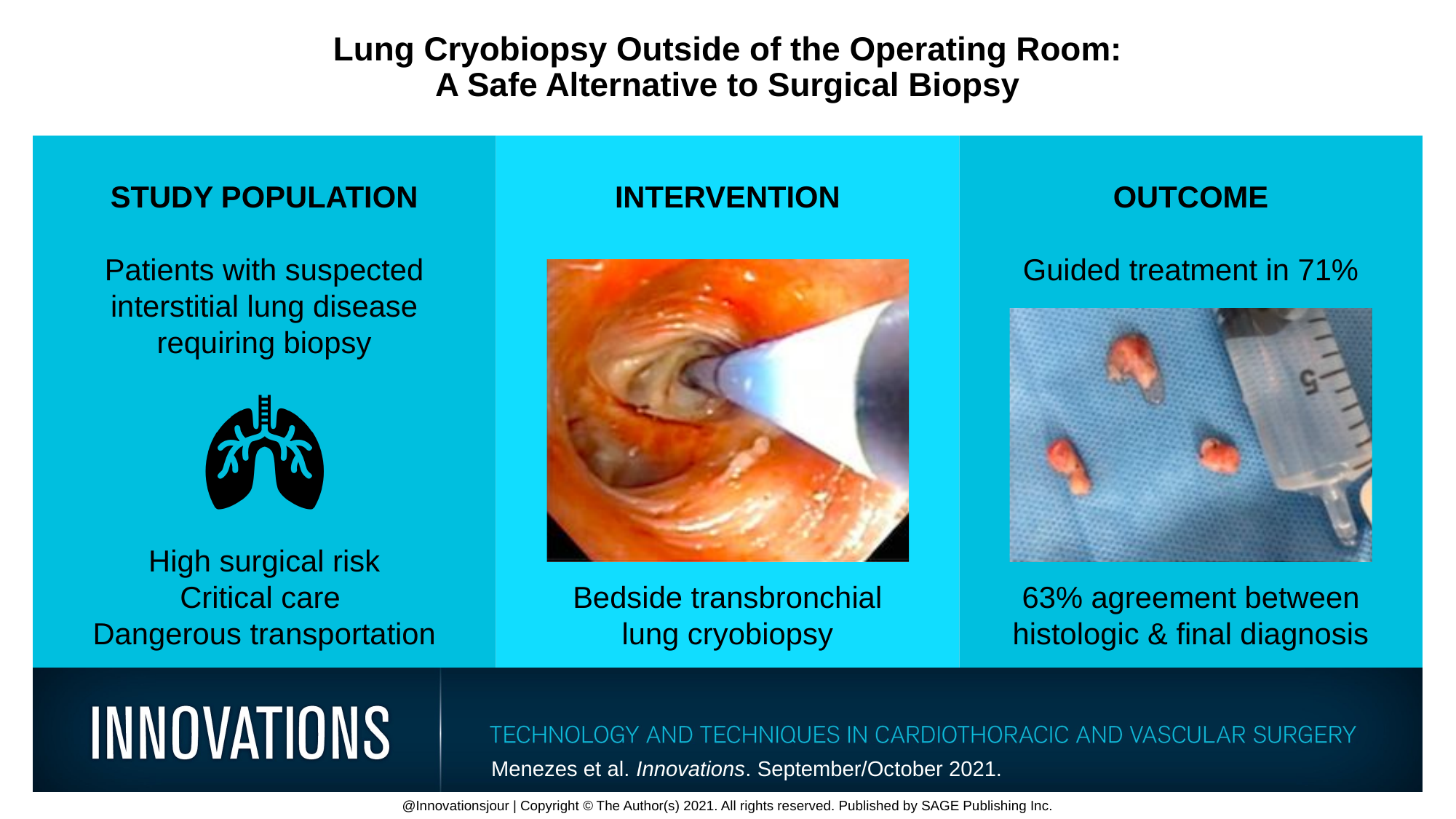

# Lung Cryobiopsy Outside of the Operating Room: A Safe Alternative to Surgical Biopsy
STUDY POPULATION
Patients with suspected interstitial lung disease
requiring biopsy
High surgical risk
Critical care
Dangerous transportation
INTERVENTION
Bedside transbronchial
lung cryobiopsy
OUTCOME
Guided treatment in 71%
63% agreement between histologic & final diagnosis
Menezes et al. Innovations. September/October 2021.
@Innovationsjour | Copyright © The Author(s) 2021. All rights reserved. Published by SAGE Publishing Inc.
